# Designing pulmonary arterial hypertension trials for detecting change in right ventricular function using cardiovascular magnetic resonance: what is the appropriate sample size?

**DOI:** 10.1186/1532-429X-15-S1-P280

**Published:** 2013-01-30

**Authors:** Karima Addetia, Nicole M Bhave, Benjamin H Freed, Mardi Gomberg-Maitland, Wendy Tsang, Victor Mor-Avi, Lira Palen, Kirk Spencer, Karin Dill, Roberto Lang, Amit R Patel

**Affiliations:** 1 Cardiology, University of Chicago, Chicago, IL, USA

## Background

Right ventricular (RV) failure is a major complication of pulmonary arterial hypertension (PAH). Cardiovascular magnetic resonance (CMR) can accurately quantify RV volume and function. Short-term changes in CMR measurements of RV size and function in PAH patients on individualized therapy have not been extensively studied; therefore, the required sample size for detecting a certain change in RV size and function in response to therapy is unknown. This study was designed to (1) assess changes in RV size and function in patients on individualized PAH treatment, and (2) to estimate sample sizes needed to detect a change in RV function in future PAH drug trials without discontinuing standard therapy.

## Methods

Nineteen patients with category I PAH were prospectively recruited. Patients were imaged using a 1.5-T scanner at baseline and after 6 months. Retrospectively gated steady-state free precession short axis cines were used to measure RV end-diastolic volume index (RVEDVI) and ejection fraction (EF) by Simpson's method of disks. Both sets of CMR data were analyzed by 2 independent observers, whose measurements were averaged. Paired, 2-sided t-tests were performed to compare end-diastolic volumes and EFs at baseline and follow up. Sample size calculations to detect changes in RVEDVI and EF over time were performed using Stata software.

## Results

Clinical characteristics and baseline measurements are shown in Table 1. Although after 6 months, 53% had an increase in EF≥3% and 26% an increase in EF≥5%, in the overall cohort, there was no significant difference over time in either mean RVEDVI (ΔEDVI 3±17ml/m2, NS) or mean RVEF (ΔEF 1±4%, NS) (Figure). To detect a 5% difference in ΔEF between a group of patients receiving standard + new treatment versus a group receiving standard treatment + placebo (90% power, α=0.05), 22 patients in each group would be required (or to detect a 3% difference, 59 patients per group). Detecting a 20ml/m2 change in RVEDVI would require 16 patients per group (or 61 patients per group to detect a change of 10ml/m2).

**Table 1:** Baseline characteristics

Characteristic	(N=19)
Age (years): average ±SD (range) % Female Body surface area(m2): average ±SD	52±11 (28-67) 95% 1.8±0.2

WHO functional class	

I (%) II (%) III (%) Unknown (%)	32% 37% 26% 5%

Past medical history	

None Hypothyroidism Atrial fibrillation Other (hypertension, stroke, asthma)	10 (53%) 6 (32%) 1 (5%) 5 (26%)

Etiology of pulmonary hypertension	

Idiopathic Congenital Connective tissue disease/scleroderma Other (HIV, anorexigen use)	7 (37%) 4 (21%) 3 (16%) 5 (26%)

Therapy for pulmonary hypertension	

No therapy Single-therapy Multi-therapy (2 or more agents)	2 (11%) 6 (32%) 11 (58%)

Baseline right ventricular size and function measurements (means)	

RVEF (%) RVEDVI (ml/m2) RVESVI (ml/m2)	35±12 136±41 91±38

SD = standard deviation; WHO, World Health Organization; RVEF, right ventricular ejection fraction; RVEDVI, right ventricular end-diastolic volume index; RVESVI, right ventricular end-systolic volume index

**Figure 1:**
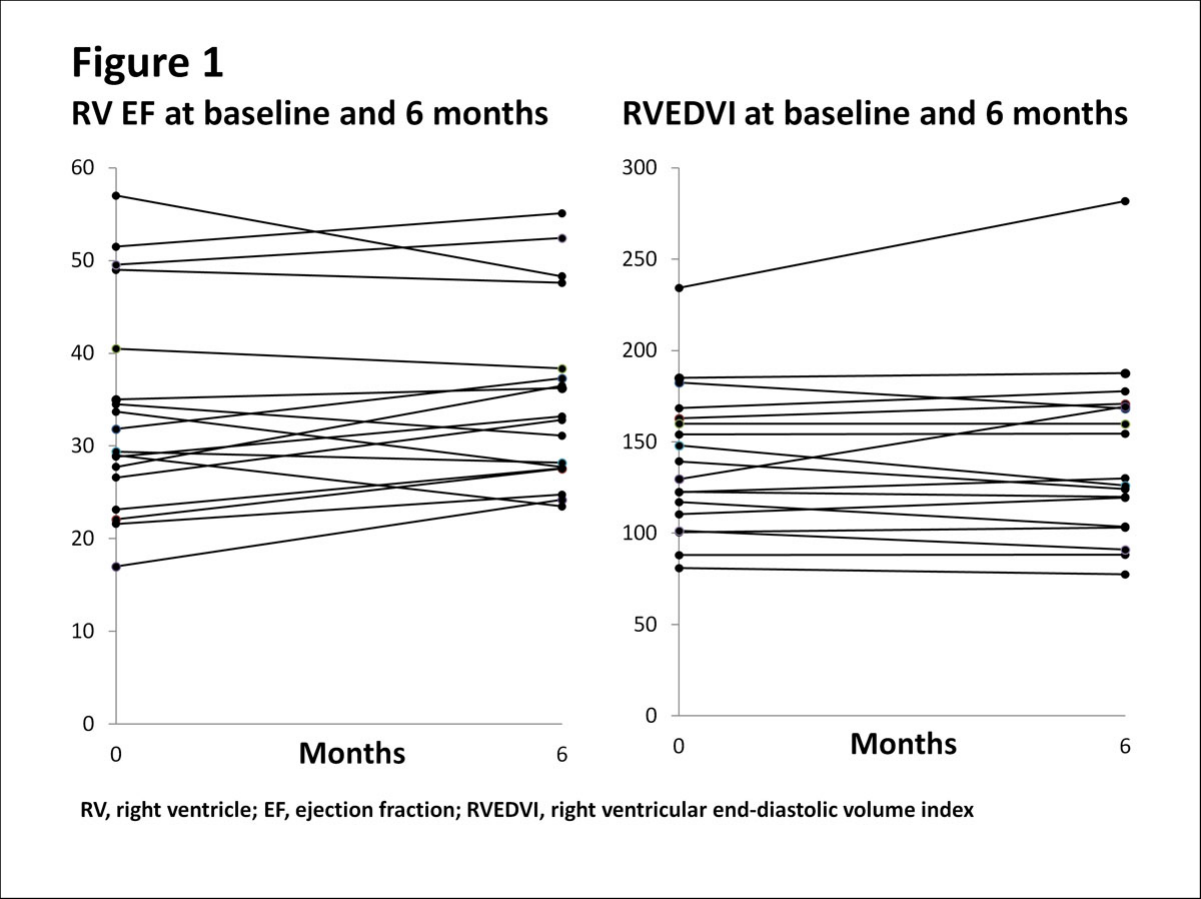


## Conclusions

CMR can be used to detect the effects of new PAH therapies on RV volume and function with relatively small groups of patients.

## Funding

None

